# Utilizing Human-in-the-Loop AI Predictions to Prescribe Personalized Falls Prevention Programs

**DOI:** 10.1093/geroni/igaf122.4313

**Published:** 2025-12-31

**Authors:** Mahederemariam Dagne, Nathan Green, Diane Murphy, Patricia Heyn

**Affiliations:** Marymount University, Arlington, Virginia, United States; Marymount University, Arlington, Virginia, United States; Marymount University, Arlington, Virginia, United States; Marymount University, Arlington, Virginia, United States

## Abstract

Falls are the leading cause of fatal and nonfatal injuries in older adults, with over 14 million falls reported annually (CDC). In response, the Administration for Community Living (ACL) launched seventeen Evidence-Based Falls Prevention Programs (EBFPPs) nationwide. From 2013 to 2024, demographic, pre-, and post-program data on 203,838 adult participants were collected. Given the robustness of this dataset, we believe AI could be used to evaluate EBFPPs, identify patterns to predict falls risk, and determine which programs work best for different individuals. This enables a more personalized, precise, and effective fall prevention strategy. Therefore, we analyzed ten key outcomes from the surveys, such as falls reduction, balance, perceived strength, and fear of falling (FoF). Changes in falls, strength, and balance were statistically significant (p < 0.05 in most pairwise tests) and showed the highest improvement in participants enrolled in FallScape (39.9%, N = 160), CAPABLE (36.2%, N = 71), Matter of Balance (34.38%, N = 41,515), and Stepping On (32.7%, N = 12,362). These results may be due to the programs’ focus on combining behavioral and exercise training rather than relying on a single modality. However, SAIL and EnhanceFitness ranked highest in reducing FoF. Based on these findings, we developed an AI-based algorithm to identify key falls-related factors, such as health status, strength, and FoF, and provide customized prevention recommendations that incorporate participant-identified priorities. Rather than adopting a one-size-fits-all approach, our method emphasizes tailoring fall interventions through human-in-the-loop AI predictions. This personalization aims to better address multiple risk factors and their combined effects on falls prevention.

